# All day-long: Sticklebacks effectively forage on whitefish eggs during all light conditions

**DOI:** 10.1371/journal.pone.0255497

**Published:** 2021-08-02

**Authors:** Jan Baer, Sarah Maria Gugele, Joachim Bretzel, J. Tyrell DeWeber, Alexander Brinker

**Affiliations:** 1 Fisheries Research Station Baden-Württemberg, Langenargen, Germany; 2 Institute for Limnology, University of Constance, Konstanz, Germany; University of Waikato, NEW ZEALAND

## Abstract

The three-spined stickleback *Gasterosteus aculeatus* invaded Lake Contance in the 1940s and expanded in large numbers from an exclusively shoreline habitat into the pelagic zone in 2012. Stickleback abundance is very high in the pelagic zone in winter near the spawning time of pelagic whitefish *Coregonus wartmanni*, and it is hypothesized that this is triggered by the opportunity to consume whitefish eggs. Field sampling has qualitatively confirmed predation of whitefish eggs by stickleback, but quantification has proven difficult due to stormy conditions that limit sampling. One fundamental unknown is if freshwater stickleback, known as visual feeders, can successfully find and eat whitefish eggs during twilight and night when whitefish spawn. It is also unknown how long eggs can be identified in stomachs following ingestion, which could limit efforts to quantify egg predation through stomach content analysis. To answer these questions, 144 individuals were given the opportunity to feed on whitefish roe under daylight, twilight, and darkness in controlled conditions. The results showed that stickleback can ingest as many as 100 whitefish eggs under any light conditions, and some individuals even consumed maximum numbers in complete darkness. Furthermore, eggs could be unambiguously identified in the stomach 24 hours after consumption. Whitefish eggs have 28% more energy content than the main diet of sticklebacks (zooplankton) based on bomb-calorimetric measurements, underlining the potential benefits of consuming eggs. Based on experimental results and estimates of stickleback abundance and total egg production, stickleback could potentially consume substantial proportions of the total eggs produced even if relatively few sticklebacks consume eggs. Given the evidence that stickleback can feed on eggs during nighttime spawning and may thereby hamper recruitment, future studies aimed at quantifying actual egg predation and resulting effects on the whitefish population are urgently needed.

## Introduction

The three-spined stickleback (*Gasterosteus aculeatus*, hereafter referred to as stickleback) is one of several aquatic invasive species in Lake Constance, one of the largest lakes in Central Europe. Sticklebacks first established in the littoral zone of Upper Lake Constance (ULC) in the 1940s [1], but by the end of 2012 had expanded from an exclusively shoreline habitat into the pelagic zone [2]. Hydroacoustic surveys conducted twice yearly in the pelagic zone from 2009 to 2018 showed an exponentially increasing population of small fish (presumably sticklebacks) starting in 2012 and plateauing after 2014 with fluctuations between 1 280 and 7 990 individuals/ha [2]. Recent trawling surveys found that stickleback density in the pelagic zone was highest in late summer (exceeding 10 000 individuals per hectare), when post-spawning and newly hatched juveniles exit the littoral zone [3]. A second peak, with densities of up to 2 000 individuals per hectare, also occurred in winter during the spawning season of the endemic and economically important pelagic whitefish *Coregonus wartmanni* (hereafter referred to as whitefish), [3]. Furthermore, hydroacoustic surveys conducted in November 2020 showed large, dense swarms of small fish that were most likely sticklebacks (around 10.000 individuals in areas less than one hectare) near larger fish that were most likely whitefish spawner stock (Baer, own observation). Since densities of zooplankton, the pelagic sticklebacks’ main diet [1], are very low and sticklebacks would otherwise starving at this time of year, it is hypothesized that this winter abundance peak might represent a migration to feed on whitefish eggs [3]. The resulting egg predation pressure could then partly explain the severe declines in whitefish recruitment observed in recent years [4].

In contrast to the clear evidence of stickleback predation on whitefish larvae in Lake Constance [1], the extent and magnitude of egg predation and its implications for whitefish recruitment are still unknown. Field investigations are especially challenging because the whitefish spawning time is difficult to predict, lasts only a few days [5], and is often accompanied by strong winds that make catching enough stickleback with gillnets or trawling difficult. Such sampling has been tried during several recent spawning seasons (several gillnets with mesh sizes from 6, 8, 10, and 12 mm set for at least five nights), but sticklebacks were only successfully captured in one year (2016) near the spawning peak. Egg feeding was qualitatively confirmed in this sample (one stickleback of 20 analysed had 8 countable eggs in the stomach), but quantifying egg predation is not possible based on this single small sample. It is also unknown if eggs are digested too quickly to enable counting eggs in stomach contents of sticklebacks caught in overnight set gillnets. Furthermore, whitefish primarily spawn during twilight and night [5] near the surface in the middle of the lake [5] and whitefish eggs also sink relatively quickly (around 1 m min^-1^; [6]) to the bottom with depths up to 250 m [5], while freshwater sticklebacks are thought to feed in well–lit environments [7] and only forage in maximum depths of around 30 m [3]. Therefore, sticklebacks must find and consume whitefish eggs while they are still near the surface, before they sink and become unavailable. If this is not possible (because they can´t find the eggs during twilight or night) a significant impact on the reproduction of whitefish could be excluded *a priori*.

Based on the above information, a laboratory experiment was designed in which sticklebacks were given the opportunity to feed on roe from fresh caught whitefish to answer the following questions: 1. Under which light conditions can sticklebacks effectively forage on whitefish eggs? 2. How long are whitefish eggs identifiable in the stomach after consumption?

In addition, the energy content of eggs and zooplankton was measured and compared to determine if there is an energetic benefit of consuming whitefish eggs, which might explain the high winter abundance of sticklebacks into the pelagic zone. Lastly, the experimental results were combined with population and fecundity estimates to calculate the theoretical number of whitefish eggs that could be consumed by sticklebacks in Lake Constance.

## Material and methods

Live sticklebacks were caught during a monthly trawling survey in the pelagic zone of ULC shortly before the beginning of whitefish spawning season (for details *cf*. [3]). 144 unharmed individuals were selected and held in two flow-through aquaria (volume 140 L) supplied with aerated lake water. All individuals were longer than 50 mm total length (TL), which is normal during this time of the year [3]. Water temperature was maintained at 8°C, which is similar to lake temperatures when whitefish spawn. Prior to the experiments, sticklebacks were acclimatized to laboratory conditions and fed daily with frozen chironomids (*Chironomidae*). Due to the fact that during this time of the year no satiated fish could be find in the field (Gugele, own observation), feeding was suspended for 48 h before the start of the foraging experiments.

Experiments were performed in three glass tanks (volume 140 L each, supplied with the same water as the aquaria) in a room with full-spectrum artificial light (NARVA BIO vital ®, LT T8 58W/958, 350 – 750 nm). The 24 h foraging experiments were conducted on three different days under three different light treatments resembling night, twilight and day with 3 replicates each. Treatment one started during complete darkness (11 h dark, followed by 0.5 h twilight, 12 h daylight, and 0.5 h twilight), treatment two started during twilight (0.5 h twilight, followed by 11 h darkness, 0.5 h twilight, and 12 h daylight) and treatment three started during daylight (12 h daylight, followed by 0.5 h twilight, 11 h darkness, and 0.5 h twilight). Transition time between darkness, twilight, and daylight was 15 minutes. The trial illuminance in lux (lx) mirrored the average illuminance measured in ULC at the depth with the highest stickleback abundance during the whitefish spawning season (15 m water depth, see [3]). Illuminance data were kindly offered by Landesanstalt für Umwelt Baden-Württemberg (LUBW) and was 0 lx at night, 0.4 klx during twilight and 0.8 klx in daylight. To simulate night conditions, all other lights and light sources (windows, doors) were masked with opaque plastic film.

For each treatment (night, twilight and day), 48 randomly selected sticklebacks were transferred to the three glass tanks (16 sticklebacks per tank) and allowed to acclimatize for 0.5 h. Therefore, the 144 sticklebacks selected for the whole experiment were divided in three equal parts (3x48). Each treatment and tank combination was assigned their own identification number for statistical analyses. After transferring the sticklebacks to the tanks, 75 mL of fresh roe (about 4 000 eggs, egg diameter 2.2–2.4 mm), obtained from freshly caught whitefish was gently added to each glass tank by turning over a spoon filled with the eggs at the water surface. In the night treatments, one person was placed in front of the aquaria before shutting of the lights and then eggs were added after waiting five minutes for the room to become dark. In all treatments, any remaining eggs were removed with suction by a small flexible tube 0.5 h after adding the eggs (using a headlamp during night conditions). 4 sticklebacks were randomly selected from each glass tank at time points of 0.5, 3, 6 and 24 h after adding eggs, euthanized with an overdose of clove oil (1 mL L^−1^) and a gill cut, measured (fresh weight, total length), and examined to determine sex. All macroscopically visible eggs and egg integuments were then counted after opening the stomach, and the digestion state was visually described. Seven individuals containing the tapeworm *Schistocephalus solidus* were excluded from the dataset to avoid bias due to possible effects of the parasite on feeding behaviour [8]. All experiments were conducted according to the German Animal Welfare Act (TierSchG) and approved by the appropriate agency (Referat Tierschutz of Regierungspräsidium Tübingen: 3/19 G; AZ 35/9185.81–4).

The effect of light intensity on egg consumption was analysed using the following general linear mixed model (GLMM) [9]:

$$Y_{ij}=\mu +\alpha_{i}+\beta_{j}+{\left(\alpha \beta \right)}_{ij}+{\delta [\eta ]}_{i}+\varepsilon_{ij}$$ (1)

where Y_ij_ is the number of consumed eggs; μ is the overall mean, α_i_ denotes light intensity during feeding, β_j_ is the sampling time (interval between feeding and sampling), (αβ)_ij_ is the interaction between light intensity and sampling time, δ(η)_i_ is the random factor nesting sticklebacks within tanks, and ε_ij_ is the random residual error. The Tukey-Kramer HSD (honestly significant difference) test was used for *post hoc* comparisons between light intensities during feeding [10]. A similar GLMM [1] was used to test the effects of sex and TL on egg consumption, where Y_ij_ is the number of consumed eggs; μ is the overall mean α_i_ denotes TL, β_j_ is sex, (αβ)_ij_ is the interaction between sex and TL, δ(η)_i_ is the random factor nesting stickleback within tanks, and ε_ij_ is the random residual error.

The energy density of zooplankton and whitefish eggs was measured using multiple samples from Upper Lake Constance. Zooplankton was sampled with a pelagic trawl net (mesh size 60 μm) at least once per month between August and November 2014 and then frozen at -20°C (eleven trawling events, sample weights between 20 and 150 g). In December 2019, 20 fully mature whitefish spawners were caught using drift gillnets in the pelagic zone of Upper Lake Constance, and 100 eggs from each female were counted. The eggs as well as each zooplankton sample (after thawing) were homogenized using a mortar and placed into pre-weighed aluminium trays. Individual samples (component plus aluminium tray) were weighed, placed in an oven at a constant temperature of 82°C for 24 h, and dried to constant mass. The gross energy (GE) of individual samples of pelagic zooplankton (n = 11) and whitefish eggs (n = 20) was measured using an IKA™ bomb-calorimeter (C 7000). The gross energy (GE) of each composite sample was determined in the calorimeter chambers using standard procedures [11] and calculated in kJ g^−1^.

The population size of stickleback and total whitefish egg production were estimated and combined with consumption experiment results to calculate potential egg consumption by sticklebacks.: The pelagic area of ULC (defined as the area with depths > 25 m) was estimated as approximately 40 000 ha. Based on a mean stickleback density of at least 2 900 individuals per ha [2], the stickleback population was estimated to be at least 115 million. The abundance of whitefish spawners in 2014 was calculated using cohort analysis following Thomas & Eckmann (2007) [12], with an assumed annual natural mortality rate of 0.2. Whitefish abundance in 2014 was estimated because this is the most recent year when all cohorts up to age 6 have been removed through fishing (abundance of older individuals is very low) as required by cohort analysis. The number of spawning females in ages 3–6 was then estimated assuming a sex ratio of 0.5. A *von Bertalanffy* growth curve fit with samples from 2013–2015 was used to estimate the mean length of each age class in November and December. The length specific fecundity was then estimated using the linear length-fecundity regression equation y = 1551.6x – 40752 (r^2^ = 0.66), which was fit using data from 23 ripe but not yet spawning females sampled in December 2020. The total number of eggs spawned in the lake was then calculated as the product of fecundity and abundance, and spawning was assumed to occur evenly throughout a 5-day period.

All statistics were run on JMP Pro 15.2.1 (64 bit, SAS Institute).

## Results

**Fig 1:**
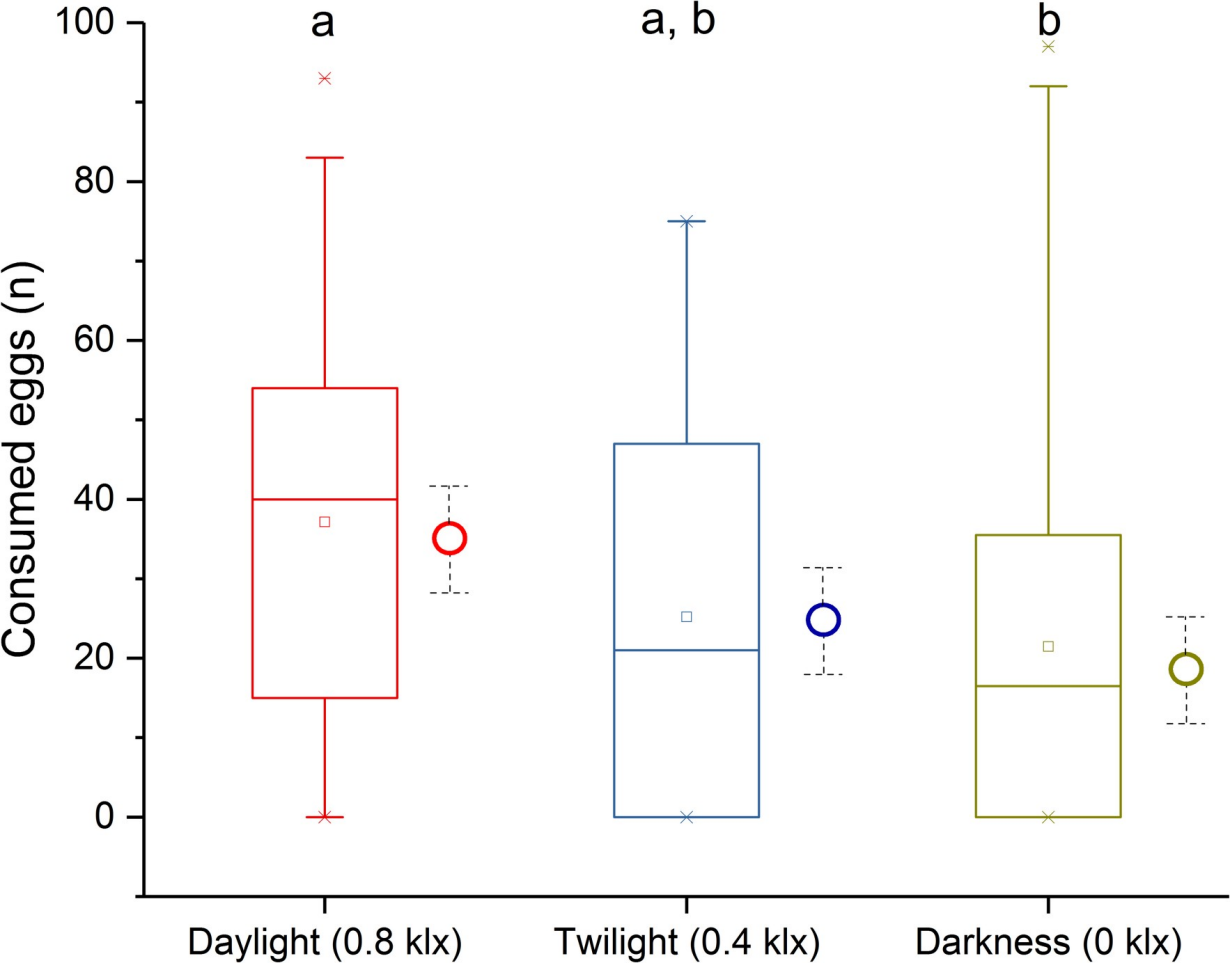
Box plot of the number of consumed eggs by light intensity treatment. Box limits indicate the 25th and 75th percentiles, centrelines show the medians, centre boxes the mean, whiskers extend 1.5 times the interquartile range from the 25th and 75th percentiles, and outliers are represented by crosses. Circles with whiskers (dotted lines) show the grand marginal means and their standard errors. Groups sharing the same alphabetic character are not significantly different (Tukey-Kramer HSD, *P <* 0.05).

In the daytime and twilight treatments, sticklebacks did not react to surface disturbances caused by introducing eggs and started to feed while eggs were slowly sinking to the bottom. Some sticklebacks consumed more than 10 eggs in a few seconds, and some sticklebacks also ate more eggs from the bottom after they had eaten sinking eggs. Across all treatments, 66.9% of all sticklebacks consumed eggs, with an average of 27.5 eggs ± 26.6 (mean ± standard deviation) per stomach. The GLMM (n = 137, d.f. = 3, r^2^ = 0.17, P = 0.02) revealed that there was no significant influence of sampling time on the number of eggs consumed (*P* = 0.12). In contrast, light intensity had a significant positive effect (*P* = 0.02). Mean egg consumption during daylight (37.2 ± 25.8) was significantly higher than during darkness (21.5 ± 25.3; *P* = 0.035, Tukey-Kramer HSD). No significant difference between twilight and the other two tested light intensities was found (Fig 1). The interaction of sampling time and light intensity during feeding also had no significant effect (*P* = 0.06).

**Fig 2:**
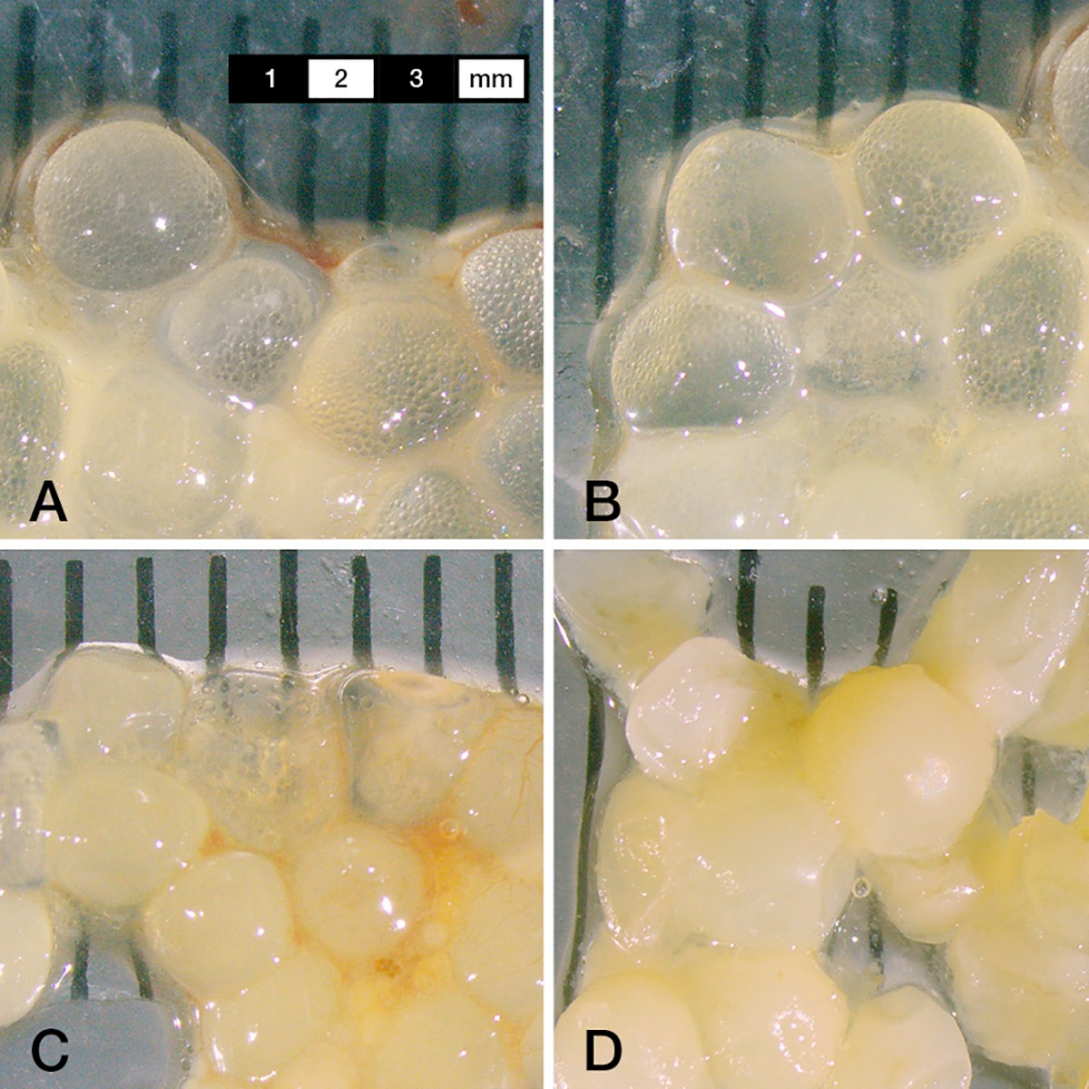
Whitefish eggs extracted from stickleback stomachs 30 minutes (A), 3 hours (B), 6 hours (C) and 24 hours (D) after eggs were available for consumption.

The mean TL of sticklebacks was 68 mm (±8 mm SD) and whitefish eggs were found in both the stomachs of the smallest (TL 55 mm) and the largest sticklebacks (TL 90 mm). The GLMM (n = 137, d.f. = 3, r^2^ = 0.13, *P* = 0.008) revealed that neither sex (*P* = 0.66), TL (*P* = 0.16), nor their interaction (*P* = 0.21) had a significant influence on egg consumption. Consumed eggs were clearly visible regardless of time sampled and were obviously swallowed whole. This was evident as only undamaged eggs with no signs of digestion were present at 0.5 and 3 h after consumption. Eggs showed the first signs of digestion 6 h after consumption, when some membranes were cracked and most showed deformations. After 24 h, all eggs were no longer circular, most were polygonal, and almost half of the eggs were cracked (Fig 2). Nevertheless, the egg membrane of cracked eggs was clearly visible and all eggs could still be counted (Fig 2).

GE of whitefish eggs (26.93 ± 0.31 kJ g^-1^) was approximately 28% higher than that of zooplankton (21.08 ± 1.29 kJ g^-1^), which was significant (t test, P < 0.05).

The estimated abundances of female whitefish spawners in 2014, mean lengths, and estimated numbers of eggs produced per age class are shown in Table 1. Total production was estimated at 2.2 billion eggs, or 440 million eggs daily if spawning occurs evenly over a 5-day period. Given an estimated population of 115 million sticklebacks, it is theoretically possible that all whitefish eggs could be consumed by stickleback if each consumed about 4 eggs daily. If only five percent of stickleback consumed 20 eggs (near the mean number of eggs consumed in dark experimental treatments) for 5 days, consumption would equal 115 million eggs or 25.8% of all eggs produced.

**Table 1:** The abundance, mean length, mean fecundity, and number of produced eggs in age classes 3–6 used to estimate total whitefish egg production in 2014.

Age	Abundance	Mean Length	Mean Fecundity	Eggs (n)
**3**	230 000	30.2	6 100	1 403 000 000
**4**	76 000	32.5	9 700	737 200 000
**5**	6 000	34.2	12 300	73 800 000
**6**	570	35.4	14 200	8 100 000
			In total	2 223 000 000

## Discussion

The results show that sticklebacks can feed on whitefish eggs during twilight and even during complete darkness under lab conditions. Consumption in twilight was not significantly lower than daylight, but consumption was 43.2% lower in the dark than in daylight. However, even in complete darkness some individuals consumed up to the maximum of 100 eggs achieved during daylight (Fig 1), which was probably near the physical restrictions of the stomach. These results are in stark contrast to the established view that freshwater sticklebacks are primarily visual predators that mainly feed in well-lit environments [7]. There is evidence that sticklebacks can feed at night in freshwater ecosystems, but only when the moon is bright enough [13]. However, nocturnal feeding has been shown in marine stickleback populations [14]. Other studies [15] also showed that visibility (turbidity) had no impact upon prey capture rates of sticklebacks, suggesting that stickleback can rely on olfactory cues or the lateral line system to successfully forage in turbid waters or perhaps even in the dark. Furthermore, new studies show that stickleback can find food using olfactory cues [16], and that combining visual and olfactory cues improves food detection [17]. In this context it should also be kept in mind that olfaction in sticklebacks is highly developed, even enabling the recognition of close relatives [18, 19]. Therefore, even if the results from lab experiments are not perfectly transferable into the wild, stickleback predation of whitefish eggs during the spawning process (during twilight and night) is certainly possible and also seems likely given their high densities (more than 10.000 individuals/ha) near spawning areas. It is expected that at least some stickleback successfully feed on whitefish eggs using visual cues in twilight and in the dark through olfaction, by detecting the movement of sinking eggs, or both. Nevertheless, follow up studies are needed to identify the mechanisms responsible for the ability of sticklebacks to feed on whitefish eggs in the dark. These studies could also determine the efficiency of stickleback foraging during complete darkness when eggs are sinking, which may help reveal mechanisms while also more closely resembling field conditions.

Whitefish eggs were clearly visible in the stomach of sticklebacks even 24 h after consumption. This long time may make field investigations easier, because eggs should be easily detected even if consumption occurred several hours or one day before sampling. Stickleback obviously swallowed the whitefish eggs whole despite possessing pharyngeal teeth [20]. Studies in brackish environments have documented sticklebacks consuming large numbers of herring eggs, which were also likely swallowed whole as they were identifiable 8 h post-feeding [21]. Digestion in our study was thus based solely on enzyme activity, which is lower in cold temperatures [22] as used here and likely helps to explain the prolonged digestion times observed. Since water temperature is similarly cold in Lake Constance when whitefish spawn, it is expected that eggs should be easily detected and counted in the stomachs of wild sticklebacks caught using gill nets fished overnight.

The potential risks for fisheries management are quite clear: given their extreme abundance in the pelagic zone, stickleback could theoretically consume a large proportion of whitefish eggs and reduce recruitment. In addition, whitefish eggs provide a very high energetic incentive compared to the main diet of sticklebacks (zooplankton). While one whitefish egg contains approximately 51 Joules (dry weight of 0.0019 g), one *Daphnia galeata*, a preferred prey of sticklebacks in Lake Constance [23], contains only about 0.5 Joules (assuming similar energy content to the zooplankton samples in this study and a dry weight of 26.1 μg [24]). Therefore, a stickleback has to consume around 100 *D*. *galeata* to get the same energy amount of one whitefish egg. The actual energetic incentive is likely even higher since zooplankton are very rare in December. These data underline the potential benefits of consuming eggs and its possible role triggering a feeding migration of sticklebacks. Our estimates suggest that even if only five percent of sticklebacks consume 20 eggs each night, around 25% of all eggs produced in 2014 would have been consumed. Such predation levels would very likely reduce whitefish recruitment and, alongside of larval predation [1], may help to explain the 50% reduction in relative abundance of small whitefish following the stickleback invasion of the pelagic zone [4]. It is tempting to speculate that stickleback could consume large proportions of whitefish eggs, especially in the littoral zone or in shallower lakes where eggs do not sink to great depths and remain available for weeks. Some caution is warranted, however, as sticklebacks did not consume eggs that were 2 days old but rather spit them back out during preliminary observations (J. Baer, unpublished data). It seems likely that the eggs are avoided after complete hardening [25], which would limit egg predation to the short time interval directly after spawning.

Given the potential implications of egg consumption, future field efforts are needed to quantify consumption of whitefish eggs by stickleback in Lake Constance. Capturing stickleback in gillnets fished overnight and analysing stomach contents should provide the necessary information since eggs are clearly visible in the stomach of sticklebacks for at least 24 hours. Larger mesh gillnets should also be fished simultaneously to capture spawning whitefish to verify that eggs were likely released nearby. As pointed out earlier, strong winds during the spawning season have limited past sampling efforts and will remain a challenge. Sufficient sampling should nonetheless be possible by fishing with a greater number of gill nets on days with suitable weather or perhaps by setting nets over 2 nights. The resulting estimates could inform future studies investigating the possible impact of sticklebacks on the whitefish population. Such studies are highly needed because the annual, pulsed mass availability of eggs could trigger specialization in stickleback through evolution as has been shown for other predators [26].

In summary, sticklebacks can forage effectively on whitefish eggs even during complete darkness. The opportunity to predate on whitefish eggs may explain why stickleback are so abundant in pelagic waters during the winter [3], especially since they are likely to benefit energetically by consuming whitefish eggs, are highly effective egg predators in other systems [21], and tend to spend more time in habitats with more profitable food sources [27]. In the future managers and scientists need to consider how whitefish recruitment may be negatively affected by egg predation, especially since only a small percentage of the huge stickleback population may consume a relatively large number of eggs.

## Supporting information

S1 File (XLSX)
